# Characterization of Fetal Antigen 1/Delta-Like 1 Homologue Expressing Cells in the Rat Nigrostriatal System: Effects of a Unilateral 6-Hydroxydopamine Lesion

**DOI:** 10.1371/journal.pone.0116088

**Published:** 2015-02-27

**Authors:** Rémy Liechti, Angélique D. Ducray, Pia Jensen, Stefano Di Santo, Stefanie Seiler, Charlotte H. Jensen, Morten Meyer, Hans Rudolf Widmer

**Affiliations:** 1 Department of Neurosurgery, Neurocenter and Regenerative Neuroscience Cluster University of Bern, Inselspital, CH-3010 Berne, Switzerland; 2 Department of Neurobiology Research, Institute of Molecular Medicine, University of Southern Denmark, Winsløwparken 21, DK-5000 Odense C, Denmark; 3 Department of Clinical Biochemistry and Pharmacology, Odense University Hospital, Sdr. Boulevard 29, DK-5000, Odense C, Denmark; 4 Department of Cardiovascular and Renal Research, University of Southern Denmark, Winsløwparken 21, DK-5000 Odense C, Denmark; Florey Institute of Neuroscience & Mental Health, AUSTRALIA

## Abstract

Fetal antigen 1/delta-like 1 homologue (FA1/dlk1) belongs to the epidermal growth factor superfamily and is considered to be a non-canonical ligand for the Notch receptor. Interactions between Notch and its ligands are crucial for the development of various tissues. Moreover, FA1/dlk1 has been suggested as a potential supplementary marker of dopaminergic neurons. The present study aimed at investigating the distribution of FA1/dlk1-immunoreactive (-ir) cells in the early postnatal and adult midbrain as well as in the nigrostriatal system of 6-hydroxydopamine (6-OHDA)-lesioned hemiparkinsonian adult rats. FA1/dlk1-ir cells were predominantly distributed in the substantia nigra (SN) pars compacta (SNc) and in the ventral tegmental area. Interestingly, the expression of FA1/dlk1 significantly increased in tyrosine hydroxylase (TH)-ir cells during early postnatal development. Co-localization and tracing studies demonstrated that FA1/dlk1-ir cells in the SNc were nigrostriatal dopaminergic neurons, and unilateral 6-OHDA lesions resulted in loss of both FA1/dlk1-ir and TH-ir cells in the SNc. Surprisingly, increased numbers of FA1/dlk1-ir cells (by 70%) were detected in dopamine-depleted striata as compared to unlesioned controls. The higher number of FA1/dlk1-ir cells was likely not due to neurogenesis as colocalization studies for proliferation markers were negative. This suggests that FA1/dlk1 was up-regulated in intrinsic cells in response to the 6-OHDA-mediated loss of FA1/dlk1-expressing SNc dopaminergic neurons and/or due to the stab wound. Our findings hint to a significant role of FA1/dlk1 in the SNc during early postnatal development. The differential expression of FA1/dlk1 in the SNc and the striatum of dopamine-depleted rats could indicate a potential involvement of FA1/dlk1 in the cellular response to the degenerative processes.

## Introduction

Idiopathic Parkinson’s disease (PD) is a chronic and slowly progressive disorder of the central nervous system, clinically defined by any combination of the cardinal motor symptoms tremor at rest, bradykinesia and muscle rigidity. The motor symptoms result mainly from the loss of dopaminergic neurons in the substantia nigra pars compacta (SNc) [1]. The etiology of sporadic PD remains unclear but is considered to consist of a complex interaction of genetic susceptibilities and environmental toxins [2]. Some studies have revealed different vulnerabilities for subpopulations of dopaminergic neurons in the SNc [3–5]. The dopaminergic neurons of the SNc mainly project to the dorsal striatum, forming the mesostriatal system, which is affected in PD. In turn the ventral tegmental area (VTA) consists of dopaminergic neurons projecting to the ventral striatum and pallidum, prefrontal cortex, amygdala, and hippocampus. This mesocorticolimbic system plays a key role in the motivational aspects of drug addiction as well as in emotional behavior [6–8].

Fetal antigen 1/delta-like homologue (FA1/dlk1) belongs to the epidermal growth factor (EGF) superfamily. Encoded by the gene*D*this protein is synthesized as a large transmembrane precursor and released from cells into circulation after proteolytic action by ADAM17 [9]. FA1/dlk1 is one of several ligands for the Notch receptor and interactions through the Notch receptor’s EGF-like repeats affect the differentiation and proliferation processes in a variety of developing cell types [10,11], including human embryonic stem cells committed to a chondrogenic lineage [12]. FA1/dlk1 acts through autocrine/paracrine and juxtacrine intercellular signaling (reviewed by [9]). Accordingly, Floridon and co-workers found that FA1/dlk1 is extensively expressed in immature cells and down-regulated during fetal development [13]. FA1/dlk1 is also involved in central nervous system differentiation [14,15], and in wound repair [16]. Moreover, FA1/dlk1 may possess tissue-specific functions in adult organs of endocrine or neuroendocrine origin [13]. In line with this, it has recently been reported that FA1/dlk1 is found in hypothalamic neurons and the authors suggest a role for FA1/dlk1 in the postnatal development of hypothalamic functions [17]. Furthermore, based on detailed immunohistochemical analyzes Meister and co-workers identified the presence of FA1/dlk1 in populations of lateral hypothalamic neurons assuming a functional role for FA1/dlk1 in orexin/hypocretin/dynorphin neurons [18]. Interestingly, FA1/dlk1 expression is seen in the midbrain of both rats and humans [15]. In addition, we identified FA1/dlk1 as a potential supplementary marker of cultured dopaminergic neurons [19] and FA1/dlk1 was found to be involved in the specification of midbrain-derived dopaminergic neurons [20]. Christophersen and co-workers demonstrated that FA1/dlk1 expression precedes the appearance of tyrosine hydroxylase (TH) in the developing mesencephalon and that FA1/dlk1 expression is induced by glial cell-line derived neurotrophic factor (GDNF). Based on the developmental expression profile of FA1/dlk1 in the SNc and the induction of FA1/dlk1 expression by GDNF, the authors concluded that the protein could play a role in mediating the regenerative and/or pharmacological effects of GDNF [21]. These latter observations are of particular interest in the context of the progressive loss of dopaminergic neurons in PD patients. The present study investigated the distribution pattern of FA1/dlk1 in the postnatal and adult rat midbrain with special attention dedicated to its association to dopaminergic neurons. Moreover, we investigated FA1/dlk1 expression in the nigrostriatal system of the 6-hydroxydopamine (6-OHDA)-lesioned rat model of PD.

Our study suggests a significant role of FA1/dlk1 in the SNc during early postnatal development. Moreover, an observed differential expression of FA1/dlk1 in the SNc and the striatum of dopamine-depleted hemiparkinsonian adult rats may reflect a potential (direct or indirect) involvement of FA1/dlk1 in the cellular response to the degenerative processes.

## Materials and Methods

### Perfusion and tissue processing

Animal experiments were performed in accordance with institutional guidelines and national regulations. The Animal Research Ethics Committee of the Canton Bern, Switzerland, and the University of Bern Animal Care and Use Committee, Switzerland approved the procedures.

Brains were collected from untreated, fluorogold (FG) injected and 6-hydroxydopamine (6-OHDA)-lesioned rats essentially as previously described [22]. Under deep pentobarbital anesthesia, postnatal day (P) 7, P14, P21 (n = 4 each, from 4 different litters) and female adult Wistar rats (n = 6) were perfused through the ascending aorta, first with a prewash solution of 0.1M phosphate-buffered saline (PBS) containing heparin (1000 I.E./100 ml, NOVO Nordisk), followed by infusion of 4% paraformaldehyde in 0.1M PBS. Immediately thereafter, the brains were removed from the skull, postfixed over night in the same fixative and cryoprotected by immersion in 15% sucrose in 0.1M PBS. After freezing in isopentane at −80°C, the brains were sectioned at 30 μm on a freezing microtome (Leica, AM1900) and the sections were mounted onto gelatine/chrom-alum and Superfrost Plus coated microscope slides or collected as free-floating sections into 24-wells plates containing anti-freeze solution (30% ethylene-glycol, 20% glycerol and 50% 0.05 M PBS, pH 7.3). For better adherence of the sections, the microscope slides were laid on a warming plate (20°C) for 5 min. and kept at −80°C until further processing.

Nissl stainings were performed for all sectioned brains to verify corresponding levels for cell analysis in striatum and substantia nigra (SN) using the atlas of Paxinos and Watson [23].

### 6-hydroxydopamine lesions

Female Wistar rats (Elevages Janvier, France), weighing 180–220 g, were anesthetized (Ketalar, 75 mg/kg, Xylapan, 5mg/kg, i.p.) and placed in a stereotaxic frame (Kopf Instruments, USA). 6-OHDA (Sigma, Switzerland) lesions were performed as described previously [22]. In brief, animals received an injection of 4 μl 32 mM 6-OHDA hydrobromide (Sigma) diluted in saline supplemented with 0.02% ascorbic acid as an antioxidant into the right striatum through a small burr hole created in the skull. The rats were randomly assigned to the following two experimental groups (n = 4 per group): group of animals sacrificed 1 week after lesion and group of animals sacrificed 4 weeks after lesion. The injection was performed over 6 min. using a 10 μl Hamilton micro syringe. The following coordinates in relation to Bregma were used: posterior 1.0 mm, lateral 3.0 mm and 5.0 mm ventral to the dura, the incisor bar was set at 0.0 mm for the striatal lesion. Animals received a subcutaneous injection of Carprofen (5 mg/kg) as postoperative analgesic. After one and 4 weeks rats were perfused as described above.

### Fluorogold injection

Female Wistar rats (Janvier Elevage, France), weighing 180–220 g, were anesthetized (Ketamin 75mg/kg and Xylazin 5mg/kg) and placed in a stereotaxic frame (Kopf Instruments, USA). For retrograde fiber tracing, animals (n = 4) were injected with 0.2μl of 0.2% fluorogold (FG) in 0.9% NaCl (Fluorochrome, LLC). The following coordinates in relation to Bregma were used: posterior 1.0 mm, lateral 3.0 mm and 4.5 mm ventral to the dura, the incisor bar was set at 0.0 mm. After injection, the needle was slowly retracted (1 mm/min). After a survival time of 10 days, the animals were reanesthetized and perfused as described above.

### Immunohistochemistry

The tissue sections were washed in 0.1 M PBS (pH 7.4) and protein blocked with 10% horse serum (HS) in 0.4% Triton X-100 PBS for 60 min. Following 3 x 5 min. in PBS brain sections were incubated overnight at 4°C with rabbit polyclonal anti-rat/mouse FA1/dlk1 (Jensen et al., 2001) diluted 1:2000 in PBS containing 0.1% Triton X-100 and 2.5% HS. After three washes in PBS the sections were incubated for 2 hrs. with biotinylated secondary antibodies (1:200, Vector Laboratories) in PBS containing 0.1% Triton X-100 and 2.5% HS. The endogenous peroxidase activity was quenched by incubation with 3.6% H_2_O_2_ and 10% methanol in 0.1M PBS for 10 min. and washed for 4 x 15 min. in PBS. For visualization of bound antibodies an avidin horseradish peroxidase complex (Vektor PK 4001, Burlingame, USA) and a metal-enhanced 3.3’-diaminobenzidine (DAB) substrate kit (Pierce, No.34065, IL, USA) was used. After rinsing in PBS and a wash in distilled water, the sections were dehydrated in ethanol, cleared in xylene, and mounted in Eukitt.

For comparative analyzes, human tissue from routine autopsies (Institute of Pathology, University of Bern Switzerland) were analyzed and processed as described above for the rat tissues. The human tissue (brain slices from the midbrain and on the level of the caudate nucleus / putamen) received immersion fixed in formalin and stored in the cryoprotection solution was from a male patient aged 70 years who suffered not from PD. Sections from the putamen were additionally stained for the cholinergic marker choline acetyl-transferase (ChAT; goat anti-ChAT antibodies at a concentration of 1:500, Millipore) as described above for the purpose of a positive control.

### Immunofluorescence staining

For co-localization studies brain sections were washed for 3 x 15 min in 0.1M PBS, pH 7.4 and incubated in 10% HS in 0.4% Triton X-100/PBS for 60 min. After a brief wash in PBS, sections were incubated overnight at 4°C with primary antibodies: rabbit polyclonal anti-rat/mouse FA1/dlk1 (1:2000, Jensen et al., 2001) in combination with mouse monoclonal anti-tyrosine hydroxylase (TH) (1:1000, Millipore); mouse monoclonal anti-calbindin (CB, 1:2000, Swant), mouse monoclonal anti-calretinin (CR, 1:2000, Swant), mouse monoclonal anti-parvalbumin (PV, 1:2000, Swant), mouse monoclonal anti-neuronal nuclei (NeuN, 1:200, Chemicon); mouse monoclonal anti-Ki-67 (1:250, BD-Biosciences); goat polyclonal anti-doublecortine (DCX, 1:100, Santa Cruz); goat polyclonal anti-dopamine and cAMP-regulated phosphoprotein (DARPP-32, 1:500, Santa Cruz); mouse monoclonal anti-glial fibrillary acidic protein (GFAP, 1:1000, Chemicon) and goat polyclonal anti-ChAT (1:500, Millipore) diluted in PBS containing 0.4% Triton X-100 and 2.5% HS. Subsequent to 3x15 min. washes in PBS, sections were incubated for 2 hrs. with either Alexa Fluor donkey anti-mouse 488nm or Alexa Fluor donkey anti-goat 488nm and Alexa Fluor donkey anti-rabbit 594nm (1:250, Molecular Probes) diluted in PBS containing 0.1% Triton-X-100 and 2.5% HS. Cell nuclei were counterstained with Hoechst 33341 (Invitrogen, Molecular Probes) at 1:10000. Thereafter, the sections were washed for 4 x 10 min. in PBS, mounted and finally covered with a solution containing 50% PBS and 50% glycerol. Fluorescence pictures were recorded using an Olympus epifluorescence microscope (BX51) equipped with a digital camera (Olympus DP72).

### BrdU detection

Following the 6-OHDA lesions, animals (n = 4) received 100mg/kg intraperitoneal injections of 5-Bromo-2’-deoxyuridine (BrdU, B5002, Sigma) diluted in 0.9% NaCl, 0.07N NaOH at a concentration of 25mg/ml. Injections started at the day when the animals were 6-OHDA lesioned and were repeated once daily for a the first week post lesion, i.e. for seven days. After 4 weeks, rats were perfusion fixed and the brains processed as described above. Brain sections were washed in 0.1M tris buffered saline (TBS), pH 7.4 and incubated in 3% H_2_O_2_ in TBS for 10 min. After extensive washes (6 x 10 min. in TBS), sections were incubated in 2N HCl for 10 min. at 37°C, reaction was stopped by incubating in 0.1M borate buffer for 10 min., followed by 6 x 10 min. washes in TBS. Sections were then incubated in 5% HS in 0.5% Triton X-100/TBS for 2 hrs., and for 4 days at 4°C with primary antibodies (mouse monoclonal anti-BrdU, 1:100, BD-Biosciences; rabbit polyclonal anti-rat/mouse FA1/dlk1 1:2000 (Jensen et al., 2001)), diluted in 0.5% Triton X-100/TBS. Subsequent to 2 x 15 min. washes in 0.5% Triton X-100/TBS and 2 x 30 min in 5% HS in 0.5% Triton X-100/TBS, sections were incubated for 2 hrs. with Alexa Fluor donkey anti-mouse 594nm and Alexa Fluor donkey anti-rabbit 488nm (1:250, Molecular Probes) diluted in 0.5% Triton X-100/TBS. Sections were then washed in PBS and mounted in PBS containing 50% glycerol for immunofluorescence observation under epifluorescence microscope (Olympus BX51) equipped with a digital camera (Olympus DP72). Digitalized images were slightly modified with the only purpose to improve quality.

### Quantification of cells

For semi-quantitative analysis of the distribution of FA1/dlk1-ir cells in the midbrain and forebrain of adult rats a bright field light microscope (Leitz Laborlux) was used. FA1/dlk1-ir cell counts were performed (40x objective) for the midbrain at the level of −5.3 mm from Bregma and for the forebrain at the level of +0.7 mm from Bregma. Only cells with an intense immunostaining and a well-preserved cell structure were included in the analyses. Immunostained brain sections were subdivided into a lattice according to the frame of reference in a microscopic ocular (each sized 0.056mm^2^). The averaged number of FA1/dlk1-ir cells per frame was then transferred into the analogous schematic image from the Paxinos Atlas. For effects of the 6-OHDA lesions on striatal FA1/dlk1-ir cells three sections (at the levels: Bregma +1.2 mm; +0 mm; −1 mm) from each of the four lesioned rats were analyzed in a blinded manner. FA1/dlk1-ir cell densities assessed in the dorsal striatum of the lesioned and contralateral unlesioned side were performed using the same method as described above. Cell counts were area corrected based on the number of frames included in the analyses and the resulting cell number was corrected according to Abercrombie [24]. Quantitative assessments of FA1/dlk1-ir cells co-expressing TH and TH-ir neurons co-expressing FA1/dlk1 in the SNc of postnatal day 7, 14 and 21 rats were performed as described in detail by Jensen and co-workers [25]. In brief, fluorescent pictures were recorded at 100x magnification using an epifluorescence microscope equipped with a digital camera. The areas of the SN were verified by the use of consecutive sections immunostained for TH using DAB. Three to six brain sections were used from each animal and cell counts were performed on both sides of the brain. TH-ir and FA1/dlk1-r cells were counted on single immunofluorescence pictures and subsequently the co-localized cells identified on the merged pictures. This allowed for verification of each co-localized cell. Co-localization rate was analyzed using Adobe Photoshop software. Furthermore, to verify the used method analyses were also done on selected sections live under the microscope. The result showed the reliability of the method used and we could exclude false identification of double labelled cells.

### Statistical evaluation

For statistical analysis of cell densities a commercially available software package (Instat, GraphPad Software) was used. Statistical comparison of cell numbers was analyzed using either the unpaired Students t-test or the two-sided non-parametric Mann-Whitney U-test. Co-localization rates of TH and FA1/dlk1 in postnatal rat brains were analyzed using ANOVA followed by the Student Newman-Keuls post hoc test. Differences were considered statistically significant at p < 0.05. Values are presented as mean ± standard error of the mean (s.e.m.).

## Results

### Expression of FA1/dlk1-ir cells in adult ventral mesencephalon

**Fig 1:**
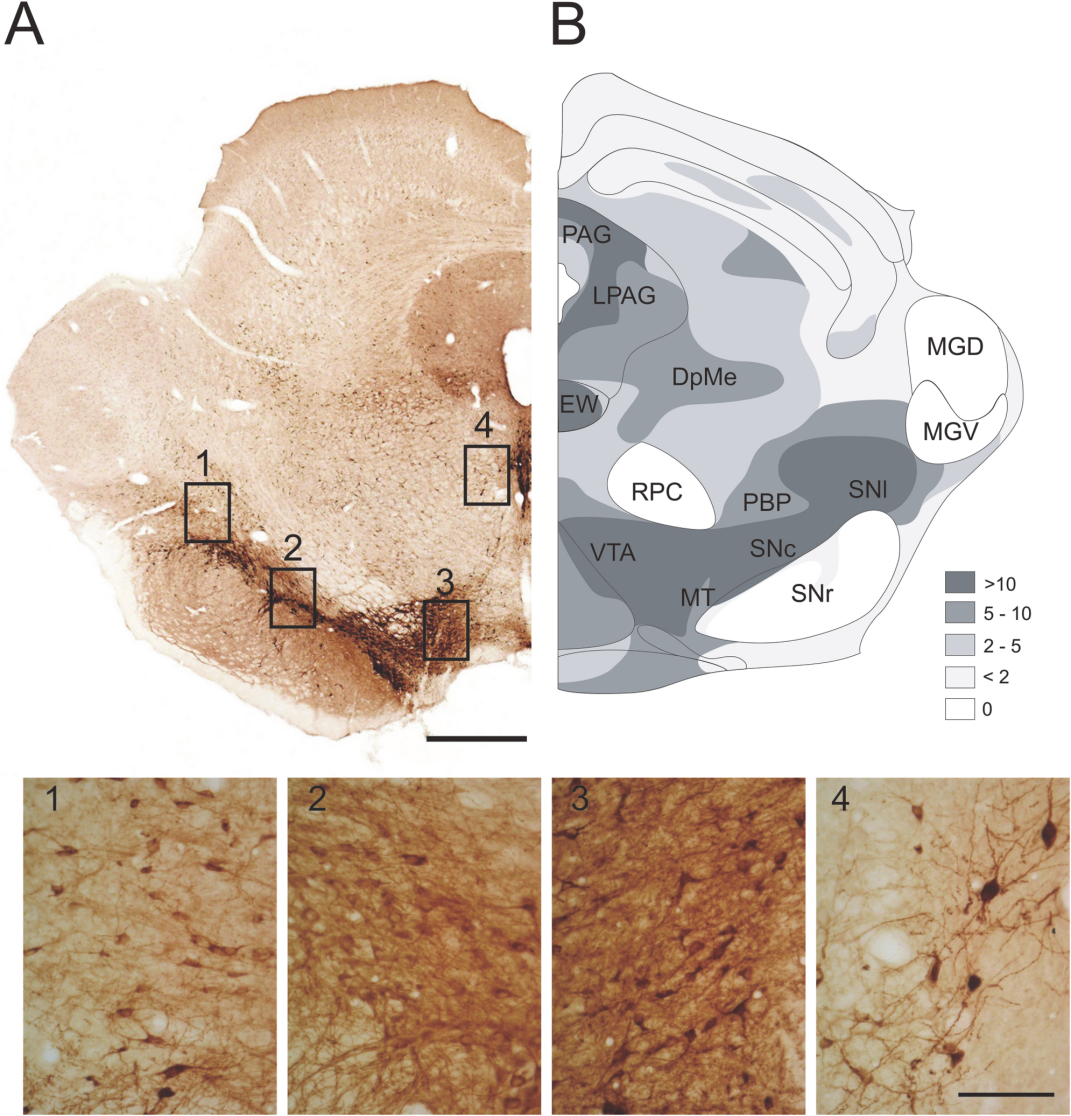
Expression pattern of FA1/dlk1 in the midbrain. Representative photomicrograph showing distinct FA1/dlk1 expression in the rat ventral mesencephalon, the lateral periaqueductal gray and Edinger-Westphal nucleus (A). Schematic drawing illustrating the distribution and density of FA1/dlk1-ir cells (see Materials and methods) (B). Photomicrographs at high magnification showing morphology of FA1/dlk1-ir cells in the substantia nigra pars lateralis (1), substantia nigra pars compacta (2), ventral tegmental area (3) and in the rostral area of Edinger-Westphal nucleus (4). Scale bars: 500μm (A, B); 100μm (1–4). Abbreviations: DpMe, deep mesencephalic nucleus; EW, Edinger-Westphal nucleus; LPAG, lateral periaqueductal gray; MGD, medial geniculate nucleus, dorsal part; MGV, medial geniculate nucleus, ventral part; MT, medial terminal nucleus of the accessory optic tract; PAG, periaqueductal gray; PBP, parabrachial pigmented nucleus; RPC, red nucleus parvicellular part; SNc, substantia nigra pars compacta; SNl, substantia nigra pars lateralis; SNr, substantia nigra pars reticularis; VTA, ventral tegmental area.

Immunohistochemical analyses revealed a significant number of FA1/dlk1-ir cells in the midbrain of adult rats. The highest density of FA1/dlk1-ir cells was found in the substantia nigra (SN), the ventral tegmental area (VTA), the deep mesencephalic nucleus (DpMe) and the periaqueductal gray (PAG) (Fig. 1). Furthermore, there was a marked FA1/dlk1 immunostaining in the area of the rostral Edinger-Westphal nucleus (EW). Within the SN, FA1/dlk1 was predominantly localized in the SN pars compacta (SNc) and pars lateralis (SNl) (Fig. 1). FA1/dlk1 expressing cells were also detected in SNc of adult human brain sections (S1 Fig.) obtained from routine autopsies (Institute of Pathology, University of Bern Switzerland, see Material and methods). Only few to none FA1/dlk1 immunostained cells were detected in the GABAergic SN pars reticulata (SNr). As depicted in photomicrographs at high magnification, several FA1/dlk1-ir cells in the SN, VTA and Edinger-Westphal nucleus showed fusiform or triangled shapes with large cell bodies (Fig. 1).

### Phenotypic characterization of FA1/dlk1-ir cells in adult rat ventral mesencephalon

**Fig 2:**
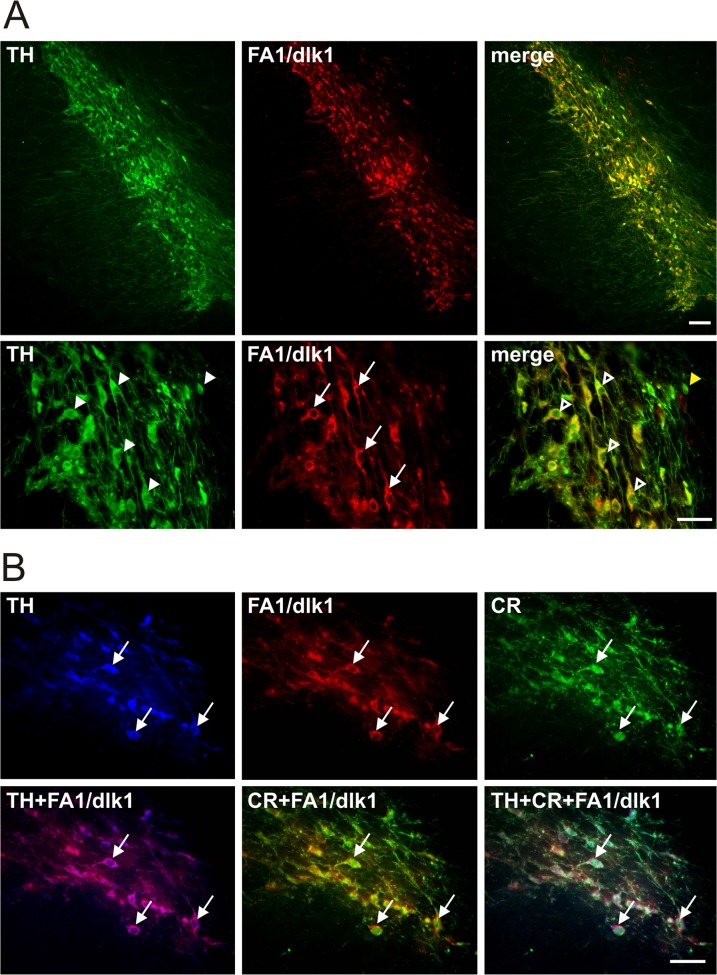
Co-expression of FA1/dlk1 with tyrosine hydroxylase and calretinin. Double immunofluorescence staining for FA1/dlk1 (FA1), tyrosine hydroxylase (TH) and calretinin (CR) in the ventral mesencephalon of adult rats. Note that nearly all FA1/dlk1-ir cells (arrows) co-localize with TH (open arrowheads), while only a very small number of TH-ir neurons did not co-express FA1/dlk1 (yellow arrowhead) (A). Triple immunofluorescence staining for FA1/dlk1, TH and CR (lower row) demonstrated co-localization of FA1/dlk1-ir cells (arrows) with both markers, TH and CR. Scale bars: 100μm (upper row in A), 50μm (lower row in A, B).

**Fig 3:**
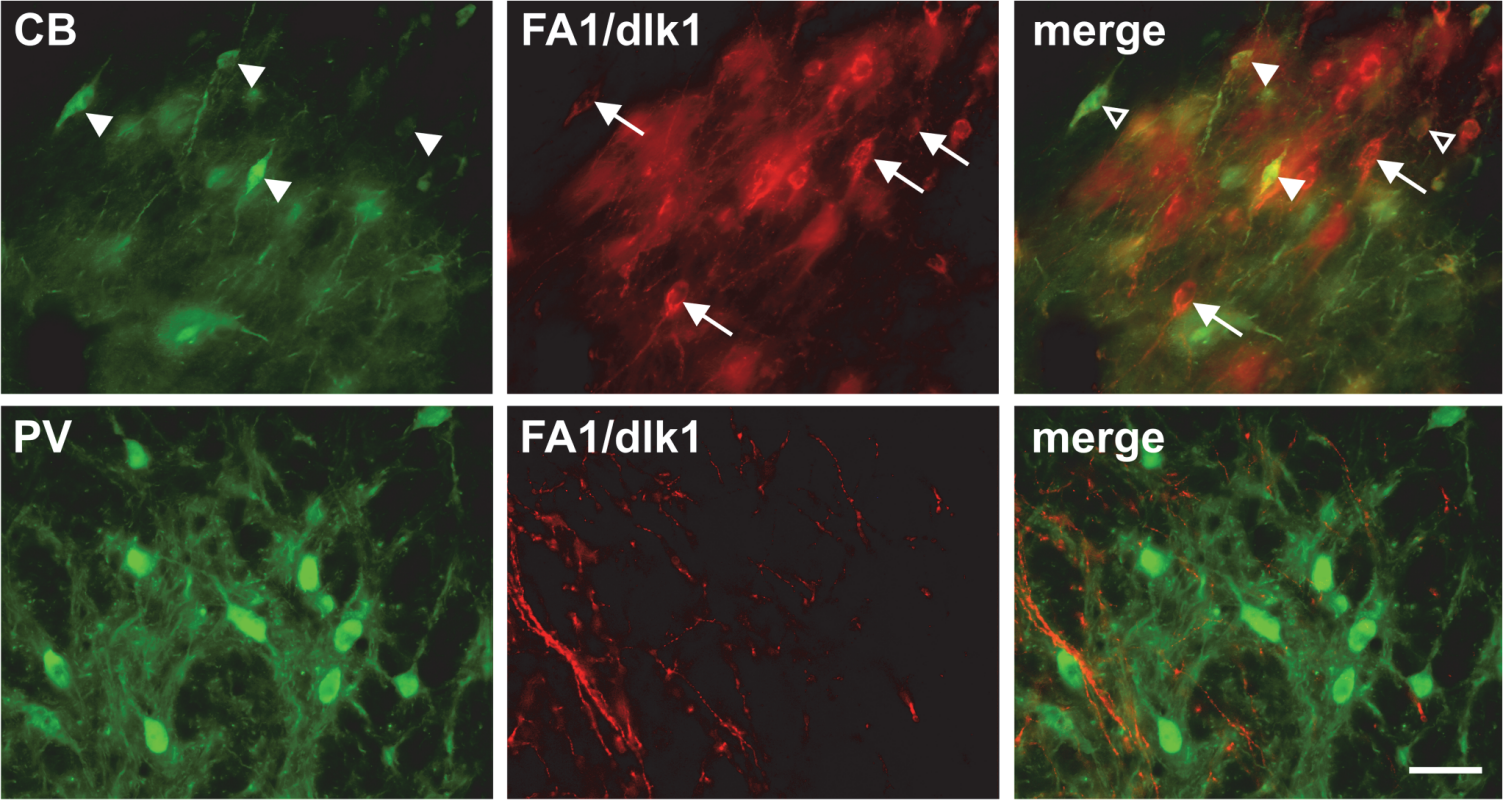
Co-expression of FA1/dlk1 with calbindin but not parvalbumin in the SN. Digitalized photomicrographs of FA1/dlk1-ir cells in the ventral mesencephalon of adult rats. Some calbindin (CB)-ir cells (arrowheads) showed co-localization with FA1/dlk1 (open arrowhead, upper row) in the substantia nigra pars compacta, whereas several CB-ir neurons (arrowheads, upper row) and FA1/dlk1-ir cells (arrows, upper row) did not co-localize. As expected from the distribution pattern depicted in Fig. 1 no co-localization was detected for FA1/dlk1 with parvalbumin (PV) in the substantia nigra pars reticulata (lower row). Scale bar: 50μm.

**Fig 4:**
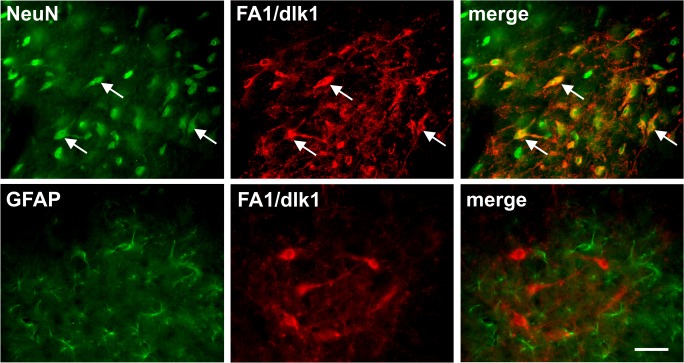
Neuronal expression of FA1/dlk1 in the SNc. Double immunofluorescence staining of FA1/dlk1 and the general neuronal marker NeuN (upper row) and the astroglial marker GFAP (lower row) in the in the substantia nigra pars compacta of adult rats. Note the distinct co-localization of FA1/dlk1 with NeuN (arrows). As expected no co-localization was found for FA1/dlk1 and GFAP. Scale bar: 50 μm.

Double immunofluorescence staining revealed that almost all of the FA1/dlk1-ir cells in the rat SNc co-localized with TH (Fig. 2A). Furthermore, only a small subgroup of TH-ir neurons did not co-express FA1/dlk1 (Fig. 2A). Triple immunofluorescence staining showed a rather high number of FA1/dlk1-ir cells co-expressing TH and the calcium-binding protein calretinin (CR) (Fig. 2B). CR-ir neurons are distributed throughout the SNc most prominently in the ventral parts [26]. We detected that 59.6 +/− 4.7% of the FA1/dlk1 positive cells co-localized with CR (n = 3 animals; mean +/− s.e.m.). A subpopulation of FA1/dlk1-ir cells showed co-expression with the calcium-binding protein calbindin (CB), which is found in neurons of the dorsal tier in the SNc [26]. (Fig. 3). We found that 19.2 +/− 2.3% of the FA1/dlk1 positive cells co-localized with CB (n = 3 animals; mean +/− s.e.m.). In accordance with the observed distribution pattern of FA1/dlk1 in the ventral mesencephalon (Fig. 1) no co-localization was detected for FA1/dlk1 and parvalbumin (PV), a calcium-binding protein expressed in a subpopulation of GABAergic neurons in the SNr [27] (Fig. 3). There was a marked co-expression of the general neuronal marker NeuN and FA1/dlk1 in cells of the SNc. In contrast, FA1/dlk1-ir cells did not co-localize with the astroglial marker glial fibrillary acidic protein (GFAP) (Fig. 4). Similarly, no co-localization for FA1/dlk1 and GFAP was detected in other regions of the brain, including the PAG and hippocampus, suggesting that FA1/dlk1 is restricted to neuronal cells (S2 Fig.).

### Co-localization of FA1/dlk1 and TH in the early postnatal SNc

**Fig 5:**
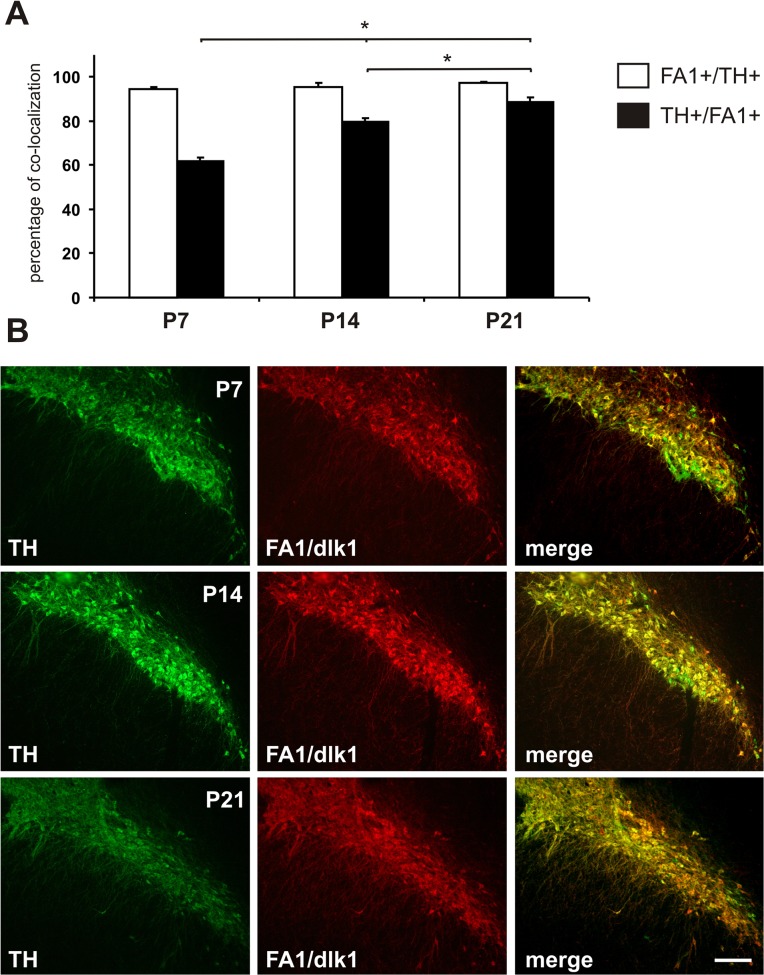
FA1/dlk1 expression rates in the developing postnatal substantia nigra. Quantification of FA1/dlk1-ir cells co-expressing tyrosine hydroxylase (TH) (open bars) and TH-ir neurons co-expressing FA1/dlk1 (filled bars) in the substantia nigra (SN) of rats at postnatal (P) day 7, 14 and 21 (A). The percentage of TH-ir neurons co-expressing FA1/dlk1 was significantly higher at P21 as compared to P7 and P14. There was no difference in the relative co-localization observed for FA1/dlk1-ir cells expressing TH during early postnatal development. Data are expressed as mean + s.e.m. *: p<0.05 vs. percentage of TH-ir neurons co-expressing FA1/dlk1 at P21. Representative digitalized pictures of double immunofluorescence staining for TH and FA1/dlk1 in SN of P7, P14 and P21 rats (B). Scale bar: 200 μm.

We found that FA1/dlk1 was expressed in cells of the SNc, SNl and VTA in the postnatal brain (S3 Fig.). Quantitative co-localization analyses revealed that more than 95% of all FA1/dlk1-ir cells in the SNc co-expressed TH at postnatal day 7 (P7), P14 and P21 (Fig. 5). At P7 out of 147.3 +/− 4.7 TH-ir and 95.2 +/− 1.3 FA1/dlk1-ir analyzed cells 90.3 +/− 1.4 showed co-localization. At P14 out of 131.8 +/− 4.6 TH-ir and 110.5 +/− 2.8 FA1/dlk1-ir analyzed cells 105.6 +/− 4.6 demonstrated co-localization. Finally, at P21 out of 144.3 +/− 8.0 TH-ir and 131.9 +/− 9.3 FA1/dlk1-ir analyzed cells 128.3 +/− 9.2 were detected to co-localize (mean +/− s.e.m.). In contrast, the percentage of the dopaminergic TH-ir cells co-expressing FA1/dlk1 in the SNc was significantly higher at P21 as compared to P7 and P14, reaching adult levels as described previously [19] (Fig. 5).

### Effects of an unilateral 6-OHDA lesion on TH and FA1/dlk1 expressing cells

**Fig 6:**
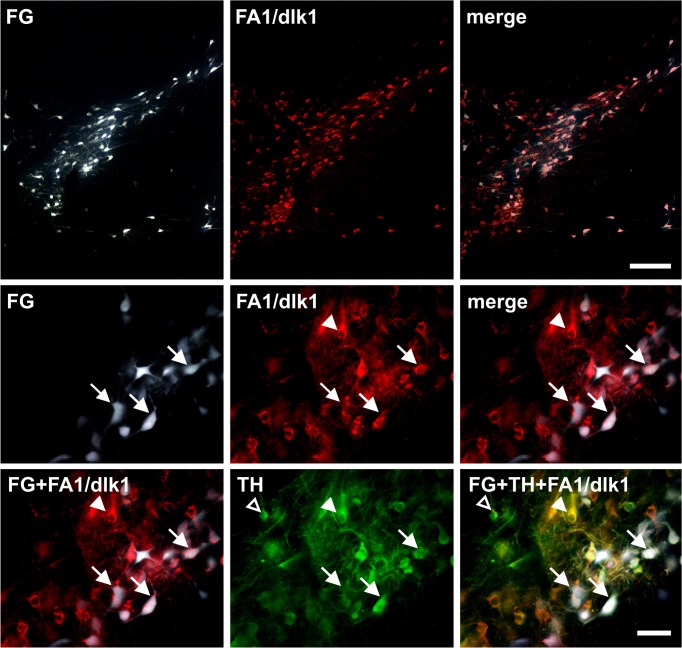
FA1/dlk1-ir cells are dopaminergic projection neurons. Representative photomicrographs of double immunofluorescence stainings showing an overview of the distribution pattern of FA1/dlk1-ir cells (FA1) and the retrograde neuronal tracer Fluorogold (FG) in rat substantia nigra pars compacta (upper row). A substantial number of FA1/dlk1-ir cells were labeled for FG (arrows, middle row). Immunofluorescence images recorded at higher magnification demonstrated that a significant number of FA1/dlk1-ir cells labeled with FG co-expressed tyrosine hydroxylase (TH) (arrows, lower row). Notably, not all FA1/dlk1-ir cells expressing TH were labeled with FG (arrowheads), and as expected some TH-ir neurons did neither label for FG nor FA1/dlk1 (open arrowheads, lower row). Scale bars: 200μm (upper row), 50μm (middle and lower rows).

**Fig 7:**
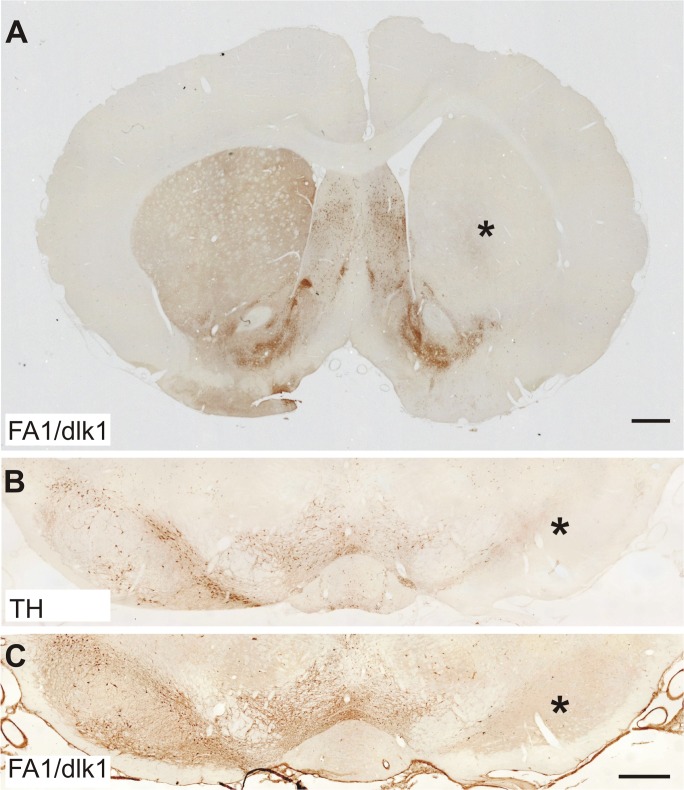
Reduced FA1/dlk1-ir cell densities in the 6-OHDA-lesioned rat brain. Representative photomicrographs demonstrating the loss of FA1/dlk1-ir fibers in the unilateral 6-OHDA-lesioned striatum (*) versus the unlesioned contralateral side (A). Enlarged photomicrographs showing the effect of the lesion on tyrosine hydroxylase (TH)-ir (B) and FA1/dlk1-ir (C) cells in the substantia nigra. Scale bars: 1mm (A); 500μm (B, C).

Retrograde fluorogold (FG) labeling and immunohistochemistry was used to investigate whether FA1/dlk1-ir cells in the SN were nigrostriatal dopaminergic projection neurons. Ten days after injection of FG into the right striatum of adult rats, FG was detected in numerous cells in the SNc. A subpopulation of the FG labeled cells co-expressed FA1/dlk1, suggesting that FA1/dlk1-ir cells in the SNc project to the striatum (Fig. 6). To further verify the role of nigral FA1/dlk1-ir cells we performed immunohistochemical analyses of FA1/dlk1 and TH in brain sections from 6-OHDA-lesioned rats (experimental model of Parkinson’s disease). As expected, the SNc was markedly depleted of TH-ir neurons on the lesioned side as assessed four weeks post-lesion (Fig. 7). Concomitantly a robust loss of striatal TH-ir fiber innervation was detected (data not shown). Similarly, we found significantly decreased numbers of FA1/dlk1-ir cells in the SNc and a marked loss of striatal FA1/dlk1-ir fibers on the dopamine-depleted side as compared to the unlesioned contralateral side (Fig. 7). Notably, FA1/dlk1-ir cells were detected in various structures including the septal nucleus, the caudate putamen (Cpu), the nucleus accumbens (Acb) and the vertical limb of the diagonal band of the forebrain of healthy control rats (S4 Fig.). In the caudate-putamen they presented with small soma sizes and were scattered over the whole striatum (S5 Fig.). A detailed analysis of the distribution pattern revealed that FA1/dlk1-ir cell densities were highest in the medial part close to the lateral ventricle and decreased gradually towards the lateral striatum (S5 and S6 Figs.). In line with the findings made for rat tissue also in the human putamen small FA1/dlk1-ir cells were detected, however, at a low number maybe due also to the poor tissue quality (S7 Fig.).

**Fig 8:**
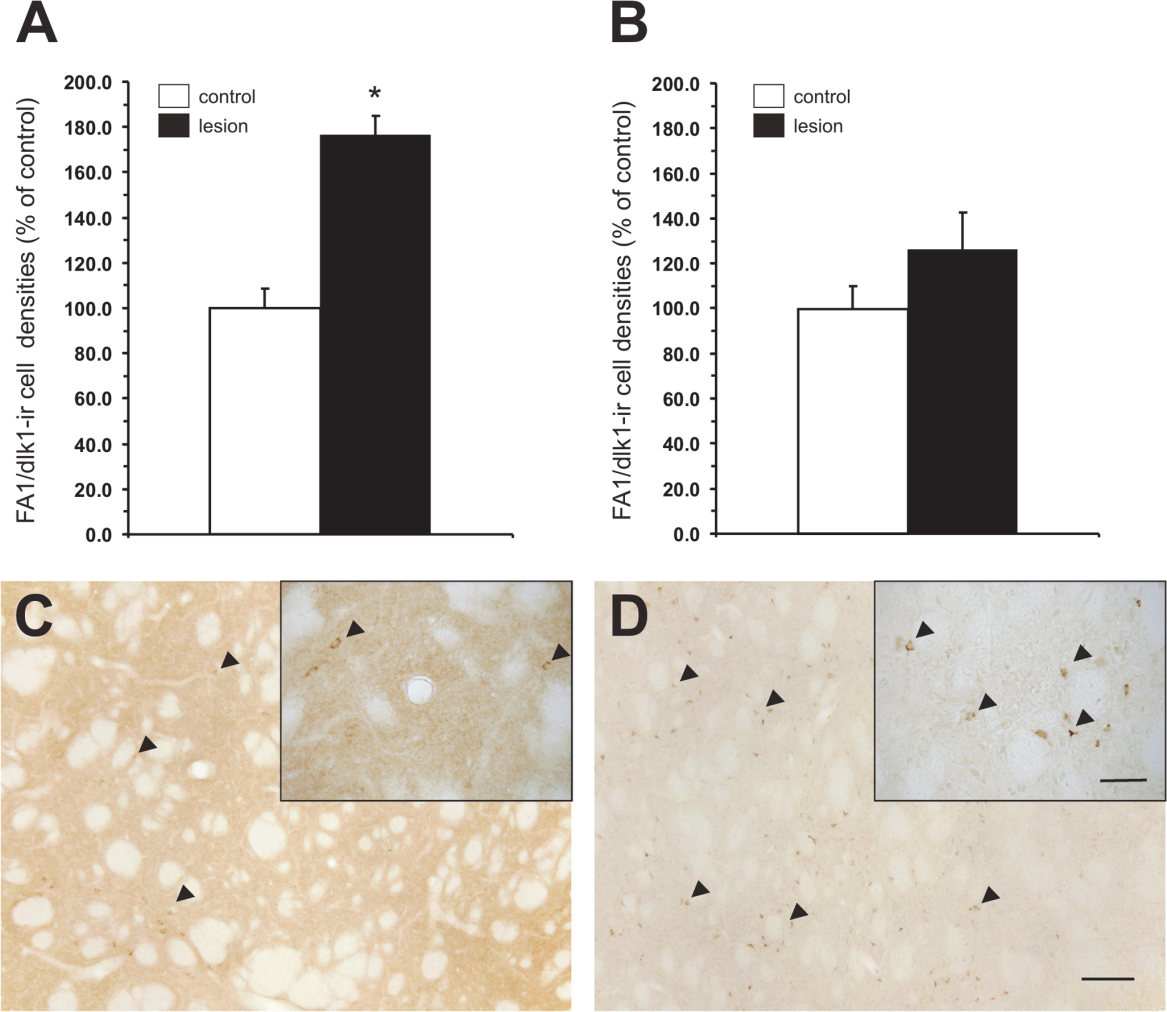
Higher FA1/dlk1-ir cell densities in the lesioned striatum. Quantitative analysis of FA1/dlk1-ir cell densities assessed in the dorsal striatum one month (A) and one week (B) after the unilateral striatal 6-OHDA lesions. Data are expressed as mean + s.e.m. and are given as percentage of corresponding controls. *: p<0.05 vs. corresponding control. Enlarged photomicrographs showing the effect of an unilateral 6-OHDA lesion on FA1/dlk1-ir cells in the striatum at the one-month time point (C, D). Rather few FA1/dlk1-ir cells (arrowheads) were observed in the unlesioned striatum (C) whereas higher densities of FA1/dlk1-ir neurons were present in the dopamine-depleted contralateral striatum (D). Scale bars: 200μm (overview); 50μm (inserts).

Interestingly, at four weeks post-lesion, we observed that the FA1/dlk1-ir cell densities were significantly higher in the denervated striatum of 6-OHDA-lesioned adult rats (57.0 +/− 2.8 cells/mm^2^; mean +/− s.e.m.) as compared to the unlesioned contralateral side (32.3 +/− 2.7 cells/mm^2^; mean +/− s.e.m.). (Fig. 8A, C, D). In order to investigate whether this increase was only seen within the severely denervated striata, FA1/dlk1-ir cell densities were also analyzed in a group of 6-OHDA-lesioned rats with progressive lesions (one week post-lesion). We found a tendency for higher FA-1/dlk1-ir cells densities on the lesioned side of these animals, however, no statistical significance could be detected (Fig. 8B).

### Phenotypic characterization of FA1/dlk1-ir cells in the lesioned striatum

**Fig 9:**
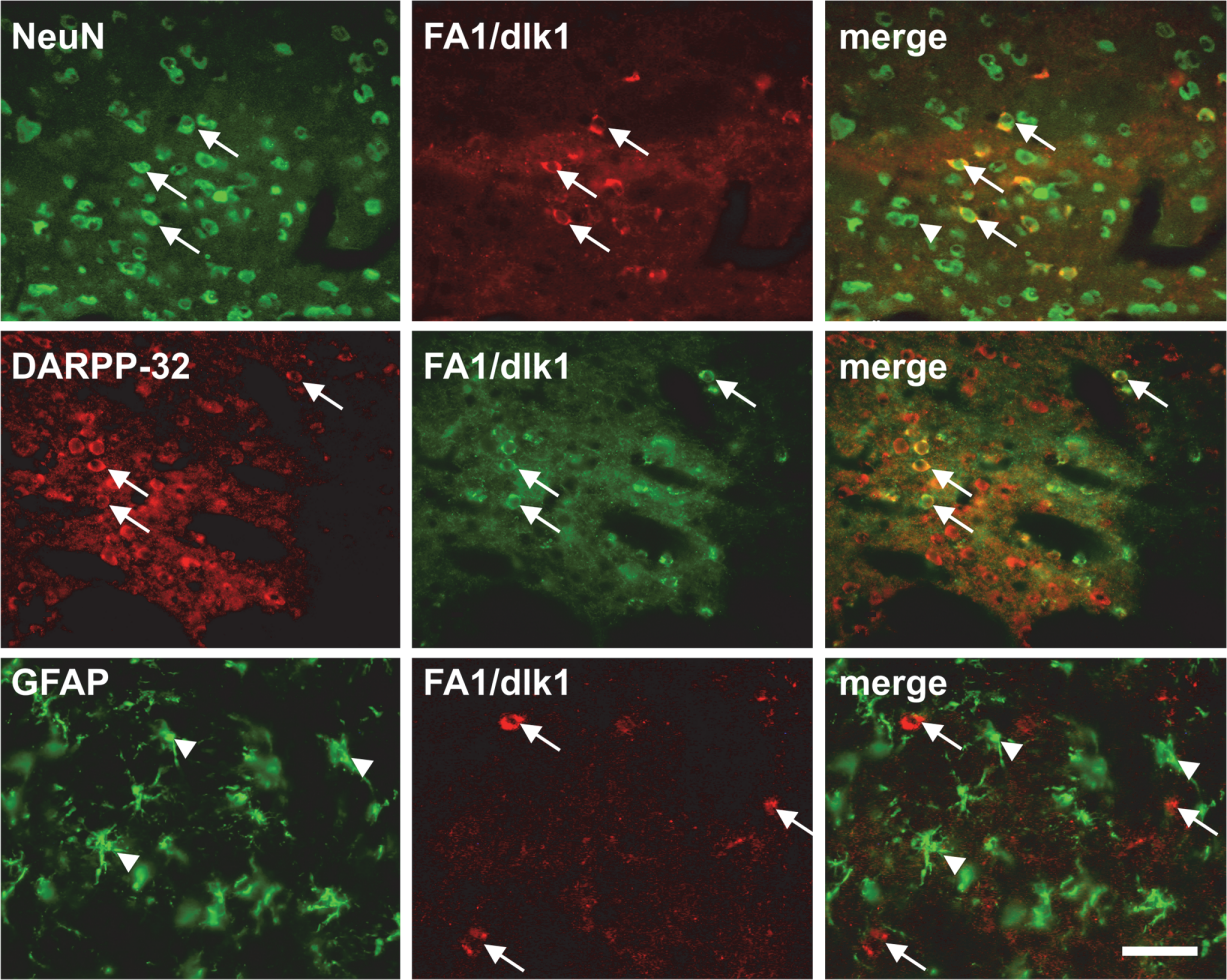
Co-localization of striatal FA1/dlk1 with neuronal markers. Representative photomicrographs of double immunofluorescence stainings demonstrating that FA1/dlk1-ir cells located in the lesioned striatum were post-mitotic neurons co-localizing with NeuN (arrows, upper row). Moreover, most of the FA1/dlk1-ir cells co-expressed DARPP-32 (arrows, middle row). FA1/dlk1-ir cells (arrows) did not co-localize with the astroglial marker GFAP (arrowheads, lower panel). Scale bar: 50μm.

**Fig 10:**
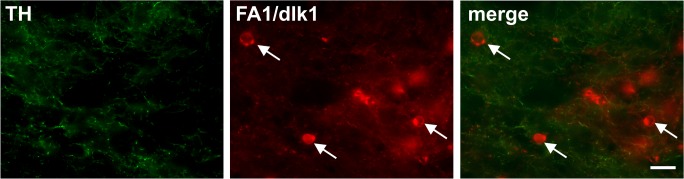
No co-localization of FA1/dlk1 with tyrosine hydroxylase in striatum. Representative photomicrographs of double immunofluorescence staining showing no co-localization of TH with FA1/dlk1 positive cells (arrows) in the lesioned striatum. Scale bar: 50μm.

**Fig 11:**
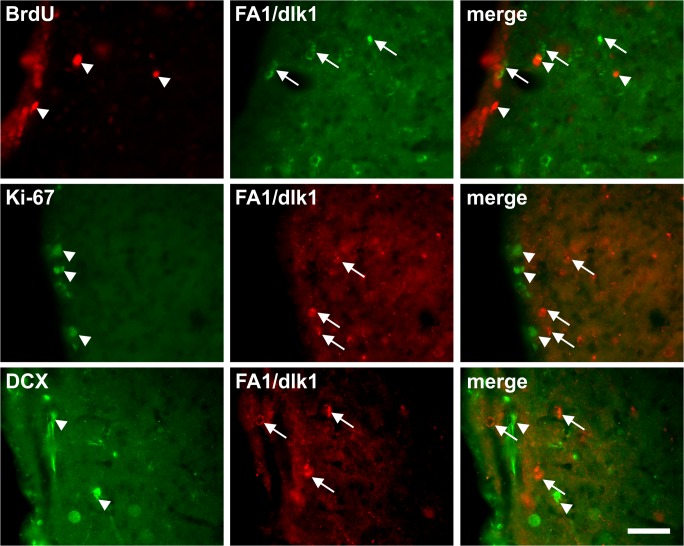
No co-localization of FA1/dlk1 with proliferation markers in SVZ. Representative photomicrographs of double immunofluorescence stainings of FA1/dlk1-ir cells in the striatum of adult rat brain. FA1-ir cells (arrows) did not co-localize with 5-bromo-2’-deoxyuridine (BrdU) (upper panel, arrowheads), Ki67 (middle panel, arrowheads) and doublecortin (DCX) (lower panel, arrowheads) in the subventricular zone and striatum 4 weeks after an unilateral intrastriatal 6-OHDA lesion. Scale bar: 50μm.

The upregulation of FA1/dlk1-ir cells in the dopamine-depleted striatum led us to further investigate the phenotype of the striatal FA1/dlk1-ir cells. A highly significant proportion (>95%) of the FA1/dlk1-ir cells co-expressed the neuronal marker NeuN, and a similar co-localization with DARPP-32 was also detected (Fig. 9). Striatal FA1/dlk1-ir cells did not co-express GFAP (Fig. 9), TH (Fig. 10) or ChAT (data not shown). Further analyses revealed that FA1/dlk1-ir cells were not newly generated neurons since no co-localization was observed for FA1/dlk1 and the proliferation markers 5-bromo-2’-deoxyuridine (BrdU) and Ki67 or doublecortin (DCX) a general marker for migrating neuroblasts/immature neurons (Fig. 11).

## Discussion

The present study shows for the first time the detailed expression pattern of FA1/dlk1 in the early postnatal and adult rat ventral mesencephalon. Notably, most of the FA1/dlk1-ir cells within the substantia nigra pars compacta (SNc) were identified as dopaminergic neurons. Moreover, we could demonstrate that FA1/dlk1 expression levels in dopaminergic neurons increases from postnatal day (P) 7 till reaching adult levels at P21. These observations may suggest a role for FA1/dlk1 during early postnatal development of the SNc. Furthermore, FG mediated retrograde tracing and immunohistochemistry identified this subgroup of FA1/dlk1-ir cells as nigrostriatal dopaminergic projecting neurons. Supporting this finding, unilateral 6-OHDA injections led to a significant loss of FA1/dlk1-ir cells in the ipsilateral SNc. Interestingly, FA1/dlk1-ir cell densities were up-regulated in the denervated striatum in response to the lesion. Interestingly, FA1/dlk1-ir cell densities were up-regulated in the denervated striatum possibly in response to the 6-OHDA-mediated loss of FA1/dlk1-expressing SNc dopaminergic neurons and / or due to the stab wound.

### FA1/dlk1 expression in adult rat midbrain

We demonstrated that FA1/dlk1-ir cells were found in high numbers in the SNc and the VTA. Similarly, FA1/dlk1-ir cells were detected in the human SNc, which is in agreement with the report from Jensen and co-workers [15]. Other midbrain regions including the deep mesencephalic nucleus, the periaqueductal gray and the rostral Edinger-Westphal nucleus disclosed also high densities of FA1/dlk1-ir cells. While our findings of high numbers of FA1/dlk1-ir cells and the robust co-localization rate with TH in the SN and the VTA support our previous view that FA1/dlk1 is a potential supplementary marker for dopaminergic neurons in these regions [19] our present data also indicate that FA1/dlk1 expression is not restricted to only dopaminergic cell populations. The observation that nearly all FA1/dlk1-ir cells in the SNc co-localized with TH is in line with our previous reports describing >90% co-localization of FA1/dlk1 and TH in the SNc of adult rats, as assessed by immunohistochemistry [19], and >80% co-localization in the SNc as assessed by combined in situ hybridization for FA1/dlk1 and immunohistochemistry for TH [15]. The dopaminergic neurons in the rat ventral mesencephalon show a distinct spatial and compartimental organization [28,29] with the cells disposed in two bands, one rostrodorsal corresponding to the SNc and one caudoventral corresponding to the SNr. The SNc can be divided into subcompartments based on the distribution of dopaminergic cell populations expressing the calcium-binding proteins CR and CB [29–31]. CR expressing cells appear relatively early in the developing rostral SN and are thereafter distributed throughout the SNc particularly in the ventral parts. CB-positive cells develop at later stages and are restricted to the dorsal SNc ([32]. In contrast, PV-ir neurons are reported to be detected in the SNr but not in the SNc [33] and are GABAergic neurons [34]. Our observation that FA1/dlk1 positive cells in the SNc co-expressed CR and to a lesser extent also CB hints to the idea that FA1/dlk1 is present in subpopulations of nigral dopaminergic neurons. Since we did not detect co-localization of FA1/dlk1 with PV in the SNr we postulate that FA1/dlk1 is not expressed in nigral GABAergic neurons. It is to note, however, that PV is not expressed over the whole SNr and that the rostromedial portion of the SNr is largely lacking PV-ir neurons but rather entirely contains CR neurons [29,33,35]. Hence, as we have not performed a detailed analysis of the co-localization pattern over the entire SNr we cannot completely exclude that FA1/dlk1 is also present in a small subpopulation of midbrain GABAergic neurons. That FA1/dlk1 is expressed in neurons is given by the morphological appearance of the cells and the observation that most of the FA1/dlk1-ir cells co-localized with the neuronal marker NeuN. In addition, no co-localization with the astroglial marker GFAP was found neither in the SN, nor in other brain regions, supporting a neuronal phenotype of FA1/dlk1-ir cells. Nevertheless, we observed that not all FA1/dlk1-ir cells co-expressed NeuN, which to some extent questions this notion. A recent study, however, reported that NeuN expression in the rat SN is variable [36] and hence it may not serve as a fully conclusive marker in the present study. Our tracing experiments with intrastriatal injections of FG furthermore revealed that FA1/dlk1-ir cells in the SNc are dopaminergic projection neurons. The observation that not all FA1/dlk1-ir cells were labeled is likely due to the circumscribed application of the FG injections in the striatum, restricting the number of nigrostriatal neurons targeted.

### Co-localization of FA1/dlk1 with TH in the postnatal SNc

The analyses of the expression pattern of FA1/dlk1 in the postnatal SNc showed that the FA1/dlk1 expression in TH-ir neurons increased from P7 to P21. This observation suggests that FA1/dlk1 may play a role during the maturation of dopaminergic neurons. While dopaminergic neurons in the midbrain are reported to appear during early embryogenesis with a peak at E13 [37,38], Park and co-workers showed that some TH-ir cells in the ependymal layer of the ventricle of the mesencephalon still undergo mitosis in the first postnatal week where after the TH-ir cell densities decrease [39]. Hence, it may be hypothesized that the higher FA1/dlk1 expression rates in TH-ir neurons reflects an increased survival of a subpopulation of dopaminergic neurons. In line with this notion is the observation that the percentage of FA1/dlk1 cells co-expressing TH remained unaffected during these developmental stages. Interestingly, it has been reported that GDNF is highly expressed in the early postnatal striatum [40,41]. Furthermore, GDNF treatment upregulated FA1/dlk1 expression levels in the midbrain suggesting that its expression precedes the appearance of TH in mesencephalic cells [21]. On the other hand, down regulation of FA1/dlk1 expression is considered an important step during differentiation in various developing cell types (for review see [42]). Studies on the possible roles of FA1/dlk1 for nigrostriatal dopaminergic neurons led to contradicting observations. Bauer and co-workers showed that supplementation of FA1/dlk1-protein to primary cultures promoted the generation of TH-ir neurons [20]. By contrast, Jacobs and co-workers analyzed the nigrostriatal system in FA1/dlk1-deficient mouse embryos during multiple developmental stages and did not observe effects on TH expression [43]. The postnatal development of dopaminergic neurons may be even more complex. We have shown that distinct subpopulations of midbrain dopaminergic neurons in the adult brain express Trefoil factor 1 (TFF1) [25]. Notably, TFF1 was expressed in dopaminergic neurons in the postnatal SNc to a similar degree as we detected in the present study for FA1/dlk1. Similarly to the findings for FA1/dlk1 the relative content of TFF1-positive cells expressing TH did not change during the first three weeks of postnatal development. In contrast to our findings with FA1/dlk1, however, the percentage of TH-ir cells expressing TFF1 was found to be down regulated during further development [25]. Taken together, additional investigations are needed to understand the role of FA1/dlk1 during postnatal development of dopaminergic neurons.

### Striatal FA1/dlk1 expression in a rat model of Parkinson’s disease

As expected, intrastriatal injection of the neurotoxin 6-OHDA resulted in a marked loss of TH-ir cells in the SNc as well as TH-ir fibers in the striatum. Similar findings were observed for FA1/dlk1-ir cells and fibers in the nigrostriatal system on the lesioned side, confirming the existence of FA1/dlk1 in dopaminergic SNc projection neurons.

Interestingly, the 6-OHDA lesions resulted in significantly higher FA1/dlk1-ir cell densities in the denervated striatum as compared to the contralateral unlesioned side. This pronounced upregulation of FA1/dlk1 seems to take place in the course and as a consequence of the progressive loss of striatal innervation after lesioning. Thus, while slightly increased FA1/dlk1-ir cell densities were detected at one-week post lesion, cell densities were significantly higher four weeks after the lesion.

A large proportion of the striatal FA1/dlk1-ir cells co-expressed the neuronal marker NeuN. In addition, many FA1/dlk1-ir cells were found to be immunopositive for DARPP-32. In the rat striatum DARPP-32 is expressed in medium-sized spiny neurons that also express dopamine D1-D5 receptors [44,45]. Thus, the high co-localization rate of FA1/dlk1 and DARPP-32 in the lesioned striatum suggests that these cells may act under influence of dopamine. In both rat and human derived tissues no co-localization was found for FA1/dlk1 and ChAT indicating that FA1/dlk1is not expressed in cholinergic interneurons. The missing co-localization of FA1/dlk1-ir cells with BrdU, Ki67 and DCX suggests that the higher cell densities were not due to neurogenesis but rather that FA1/dlk1 was up-regulated in already existing cells in response to the lesions. Nevertheless, we cannot rule out that we missed cell replication cycles using our BrdU paradigm. In addition, we cannot exclude that potentially newborn cells did not express the markers DCX or Ki67 at the time point examined. While very few TH expressing cells exist in the rat striatum under normal condition, in response to 6-OHDA lesions their numbers increase substantially. This increase in TH-positive cells has been shown to be the result of a phenotypical shift of already existing striatal cells rather than from newly generated neurons (for review see: [46]). As we did not find any TH-ir cells in the denervated striatum, which may be due to the overall low number of TH-ir cells or suboptimal staining conditions, we cannot address a potential expression of FA1/dlk1 in these cells. Further investigations are needed to elucidate the possible role of FA1/dlk1 expressing cells in the denervated striatum.

## Conclusions

Our findings demonstrate that FA1/dlk1 displays a distinct expression pattern during early postnatal development and in the adult rat brain, with marked expression in a significant subset of dopaminergic projection neurons in the SNc. A differential expression of FA1/dlk1 in the SNc and striatum of dopamine-depleted rats could indicate for an involvement of FA1/dlk1 in the cellular response to the degenerative processes.

## Supporting Information

S1 Fig Expression pattern of FA1/dlk1 in the human substantia nigra. Representative digitalized photomicrographs of sections from the adult human substantia nigra (asterisk in brain slice image; RN: red nucleus; SN: substantia nigra) in immunostained for tyrosine hydroxylase (TH) and FA1/dlk1. Note the immunoreactive cell bodies (arrows) and fibers (arrowheads). Scale bars: 300μm (brain slice image) and 100μm (photomicrographs).
(TIF)

S2 Fig No co-localization of FA1/dlk1 with GFAP in periaqueductal gray and hippocampus. Representative digitalized photomicrographs of sections from the adult periaqueductal gray (PAG) (upper row) and CA3 region of the hippocampus (lower row) immunostained for glial fibrillary acidic protein (GFAP) and FA1/dlk1. Note that no co-localization for GFAP and FA1/dlk1 was detected. Scale bars: 200μm (overview), 100μm (higher magnifications).
(TIF)

S3 Fig Expression pattern of FA1/dlk1 in the adult and postnatal midbrain. Representative digitalized photomicrographs of sections from the midbrain of adult and postnatal (P) rats at P7, P14 and P21 immunostained for FA1/dlk1. Note that at all developmental stages FA1/dlk1 immunoreactive cell bodies are detected in the ventral tegmental area (VTA), substantia nigra pars compacta (SNc) and the substantia nigra pars lateralis (SNl). Scale bar: 500μm.
(TIF)

S4 Fig Expression pattern of FA1/dlk1 in the forebrain. Representative photomicrograph showing FA1/dlk1 staining in the dorsal striatum of adult rats at the level Bregma +1mm (A). Schematic drawing illustrating the pattern of FA1/dlk1-ir cell densities. The grey level represents the density of FA1/dlk1-ir cells detected per frame (B). 1–4: Photomicrographs at higher magnification depict morphology of FA1/dlk1-ir cells in the cortex (1), nucleus accumbens (Acb) (2), subventricular striatum (3) and medial septal nucleus (MS) (4). Scale bars: A, B; 1mm, 1–4; 100μm. Abbreviations: Acb, accumbens nucleus; cc, corpus callosum; Cpu, caudate putamen; LSD, lateral septal nucleus dorsal part; LSI, lateral septal nucleus intermediate part; LSV, lateral septal nucleus ventral part; mfb medial forebrain bundle; MS, medial septal nucleus; VDB, nucleus of the vertical limb of the diagonal band; VP, ventral pallidum.
(TIF)

S5 Fig Expression pattern of FA1/dlk1 in the striatum. Representative digitalized photomicrographs showing FA1/dlk1 immunostained cell somata and fibers in the dorsal striatum at the level Bregma +1mm (A). Enlarged photomicrographs showing the scattered distribution pattern of FA1/dlk1-ir cells in the unlesioned striatum (B, C). Scale bars: A: 1mm; B, C: 50μm.
(TIF)

S6 Fig Expression pattern of FA1/dlk1 in the dorsal striatum. The detailed expression of FA1/dlk1 immunostained cell somata in the dorsal striatum of control animals revealed that higher cell densities were detected in the region in the vicinity of the lateral ventricle as compared to the lateral striatum. Data are expressed as mean + s.e.m. and are given as FA1/dlk1-ir cells per mm^2^ in the 15 areas analyzed as described in M & M.
(TIF)

S7 Fig Expression pattern of FA1/dlk1 in the human putamen nigra. Representative digitalized photomicrographs of sections from the adult human putamen (asterisk in brain slice image; ac: anterior commissure, CP: caudate nucleus; IC: internal capsule, LV: lateral ventricle, Pu: putamen) in immunostained for choline acetyl-transferase (ChAT) and FA1/dlk1. Note the low number of small FA1/dlk1-ir cell bodies (arrows) as compared to the larger ChAT-ir neurons. Numerous FA1/dlk1-ir fibers (arrowheads) were detected next to the small cell bodies. Scale bars: 500μm (brain slice mage), 100μm (overviews), 20μm (magnifications).
(TIF)
