# Supplementary figures and images for: Characterization of Fetal Antigen 1/Delta-Like 1 Homologue Expressing Cells in the Rat Nigrostriatal System: Effects of a Unilateral 6-Hydroxydopamine Lesion
Source: PLoS One. 2015 Feb 27;10(2):e0116088. doi: 10.1371/journal.pone.0116088 (PMC4344227; doi:10.1371/journal.pone.0116088)

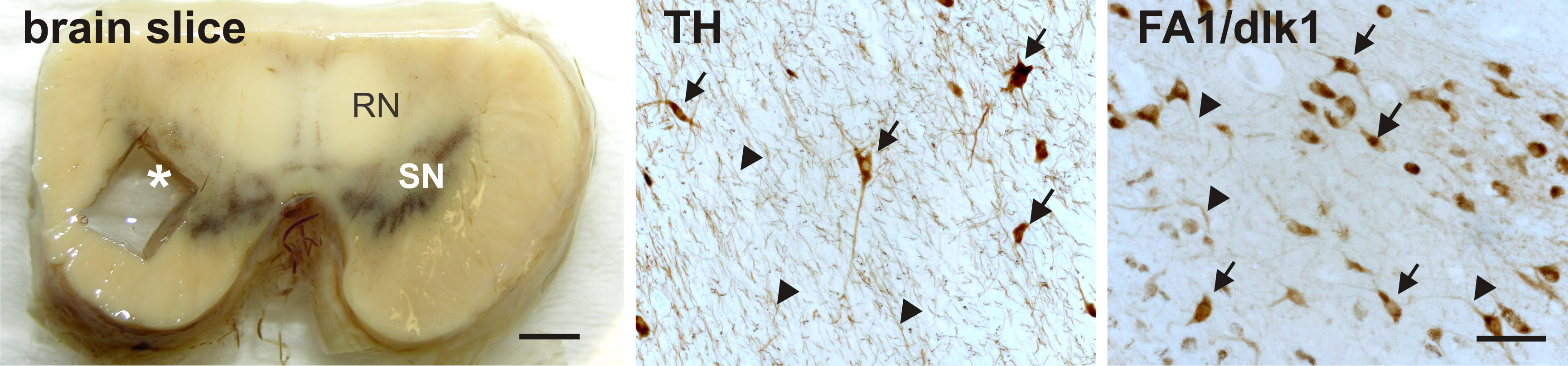

Supplement: S1 Fig — Representative digitalized photomicrographs of sections from the adult human substantia nigra (asterisk in brain slice image; RN: red nucleus; SN: substantia nigra) in immunostained for tyrosine hydroxylase (TH) and FA1/dlk1. Note the immunoreactive cell bodies (arrows) and fibers (arrowheads). Scale bars: 300μm (brain slice image) and 100μm (photomicrographs). (TIF) [file pone.0116088.s001.tif]

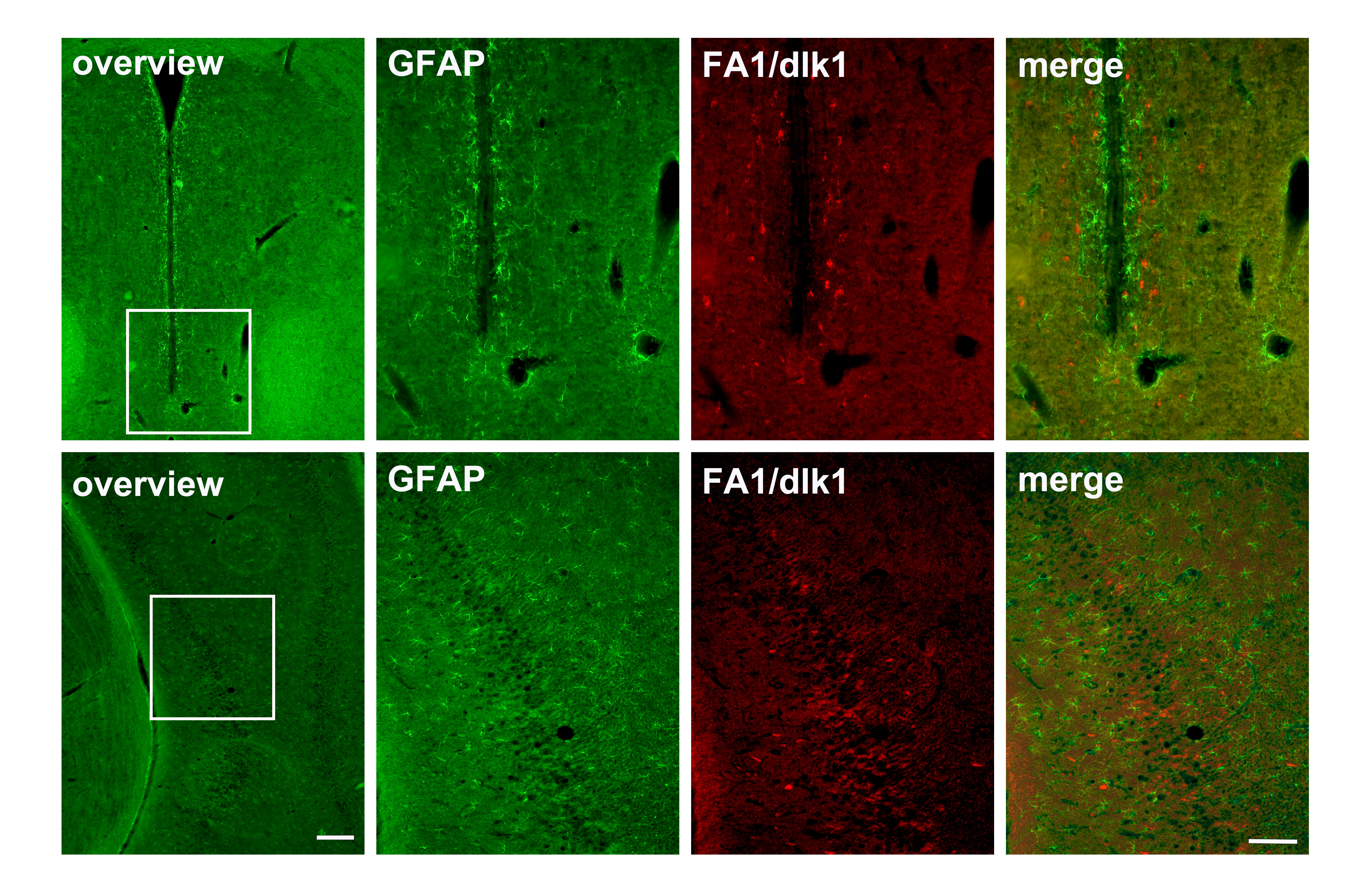

Supplement: S2 Fig — Representative digitalized photomicrographs of sections from the adult periaqueductal gray (PAG) (upper row) and CA3 region of the hippocampus (lower row) immunostained for glial fibrillary acidic protein (GFAP) and FA1/dlk1. Note that no co-localization for GFAP and FA1/dlk1 was detected. Scale bars: 200μm (overview), 100μm (higher magnifications). (TIF) [file pone.0116088.s002.tif]

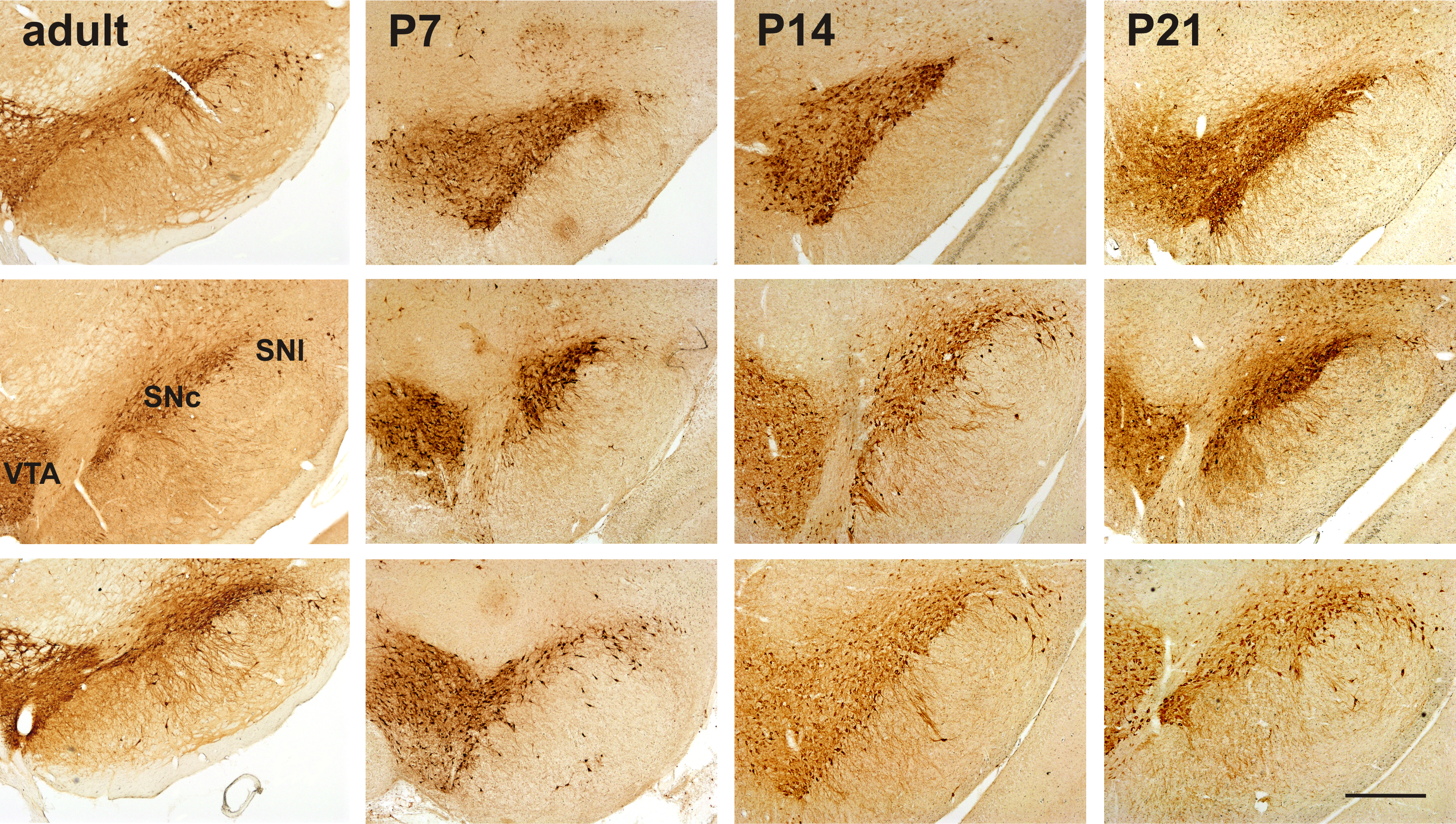

Supplement: S3 Fig — Representative digitalized photomicrographs of sections from the midbrain of adult and postnatal (P) rats at P7, P14 and P21 immunostained for FA1/dlk1. Note that at all developmental stages FA1/dlk1 immunoreactive cell bodies are detected in the ventral tegmental area (VTA), substantia nigra pars compacta (SNc) and the substantia nigra pars lateralis (SNl). Scale bar: 500μm. (TIF) [file pone.0116088.s003.tif]

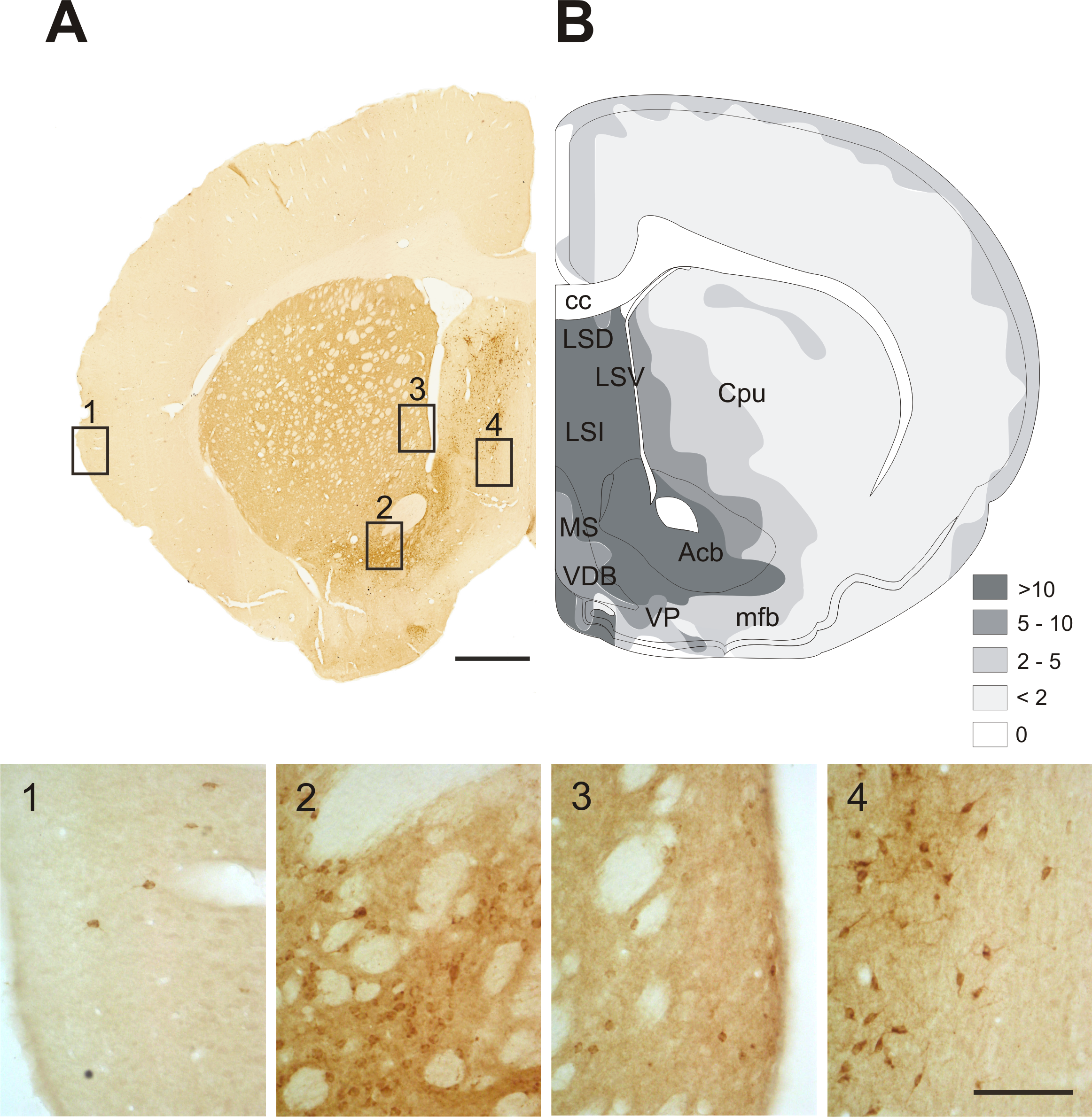

Supplement: S4 Fig — Representative photomicrograph showing FA1/dlk1 staining in the dorsal striatum of adult rats at the level Bregma +1mm (A). Schematic drawing illustrating the pattern of FA1/dlk1-ir cell densities. The grey level represents the density of FA1/dlk1-ir cells detected per frame (B). 1–4: Photomicrographs at higher magnification depict morphology of FA1/dlk1-ir cells in the cortex (1), nucleus accumbens (Acb) (2), subventricular striatum (3) and medial septal nucleus (MS) (4). Scale bars: A, B; 1mm, 1–4; 100μm. Abbreviations: Acb, accumbens nucleus; cc, corpus callosum; Cpu, caudate putamen; LSD, lateral septal nucleus dorsal part; LSI, lateral septal nucleus intermediate part; LSV, lateral septal nucleus ventral part; mfb medial forebrain bundle; MS, medial septal nucleus; VDB, nucleus of the vertical limb of the diagonal band; VP, ventral pallidum. (TIF) [file pone.0116088.s004.tif]

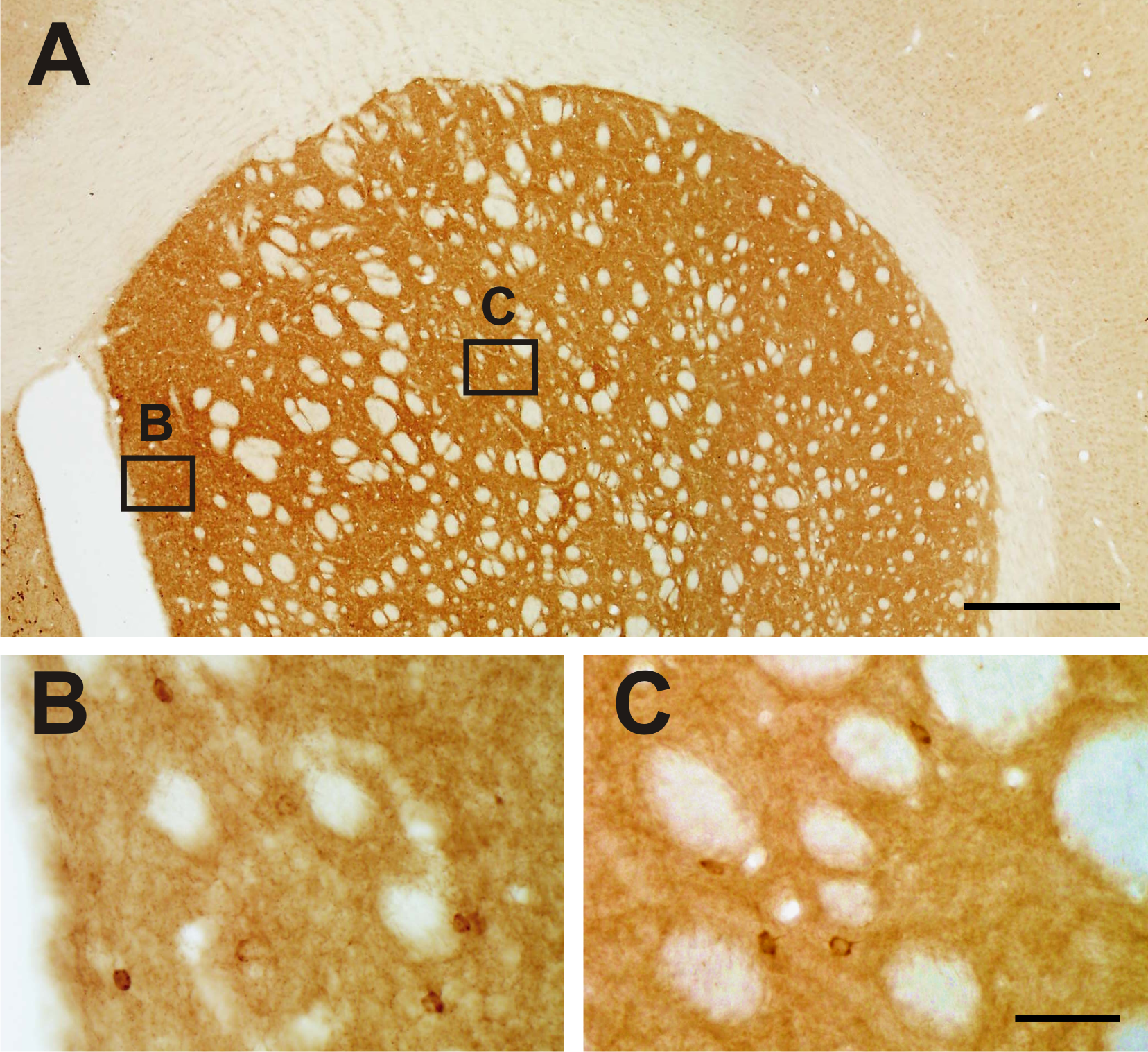

Supplement: S5 Fig — Representative digitalized photomicrographs showing FA1/dlk1 immunostained cell somata and fibers in the dorsal striatum at the level Bregma +1mm (A). Enlarged photomicrographs showing the scattered distribution pattern of FA1/dlk1-ir cells in the unlesioned striatum (B, C). Scale bars: A: 1mm; B, C: 50μm. (TIF) [file pone.0116088.s005.tif]

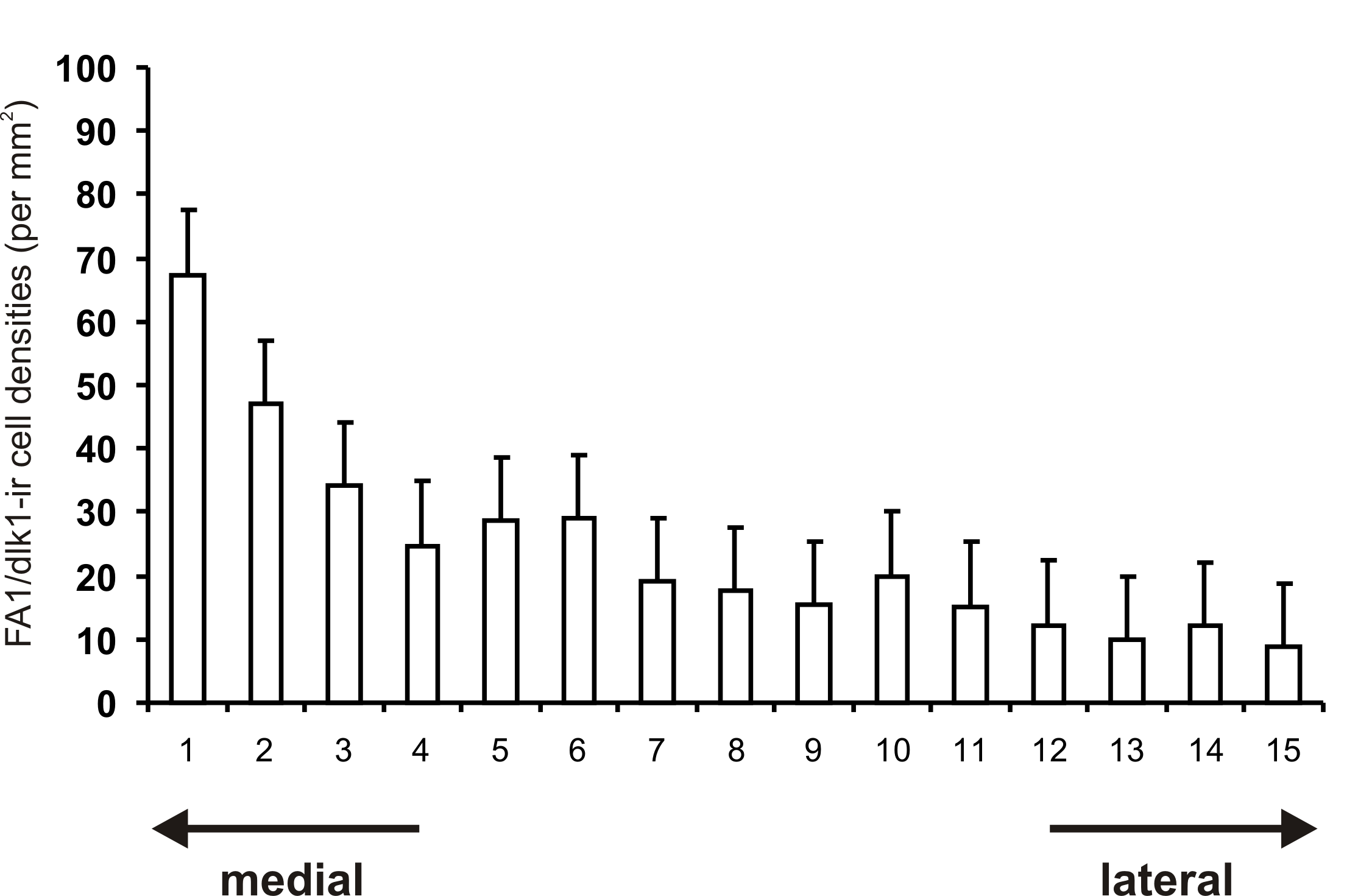

Supplement: S6 Fig — The detailed expression of FA1/dlk1 immunostained cell somata in the dorsal striatum of control animals revealed that higher cell densities were detected in the region in the vicinity of the lateral ventricle as compared to the lateral striatum. Data are expressed as mean + s.e.m. and are given as FA1/dlk1-ir cells per mm2 in the 15 areas analyzed as described in M & M. (TIF) [file pone.0116088.s006.tif]

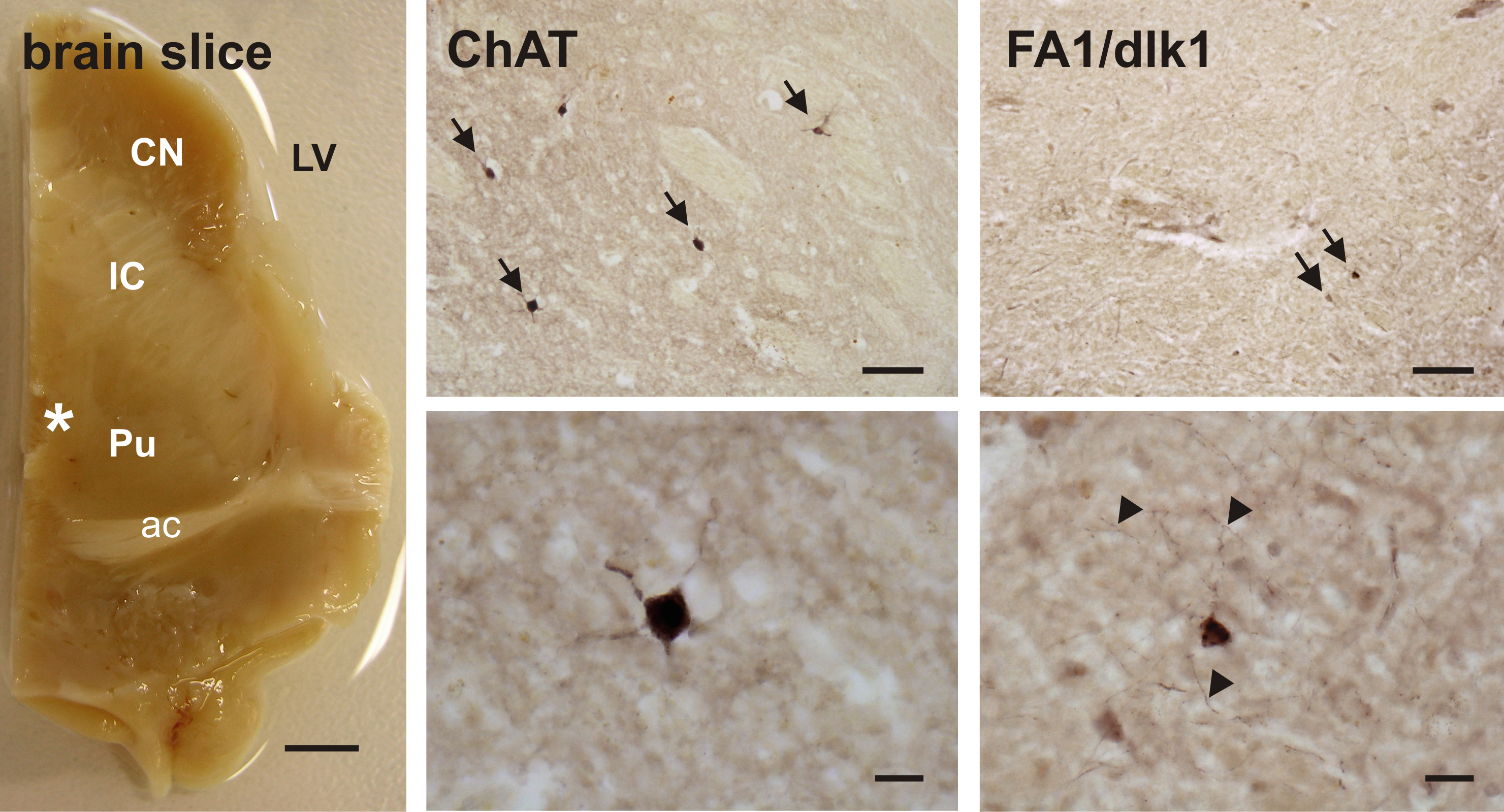

Supplement: S7 Fig — Representative digitalized photomicrographs of sections from the adult human putamen (asterisk in brain slice image; ac: anterior commissure, CP: caudate nucleus; IC: internal capsule, LV: lateral ventricle, Pu: putamen) in immunostained for choline acetyl-transferase (ChAT) and FA1/dlk1. Note the low number of small FA1/dlk1-ir cell bodies (arrows) as compared to the larger ChAT-ir neurons. Numerous FA1/dlk1-ir fibers (arrowheads) were detected next to the small cell bodies. Scale bars: 500μm (brain slice mage), 100μm (overviews), 20μm (magnifications). (TIF) [file pone.0116088.s007.tif]
